# A Lyapunov-Based Analysis on the Almost Periodicity of Impulsive Conformable Reaction–Diffusion Neural Networks with Distributed Delays

**DOI:** 10.3390/e27121246

**Published:** 2025-12-11

**Authors:** Ivanka Stamova, Gani Stamov, Cvetelina Spirova

**Affiliations:** 1 Department of Mathematics, University of Texas at San Antonio, San Antonio, TX 78249, USA; 2 Department of Mathematics, Technical University of Sofia, 8800 Sliven, Bulgaria; cvetelina2802@abv.bg

**Keywords:** reaction–diffusion neural networks, distributed delays, impulses, conformable derivatives, almost periodicity, global conformable exponential stability

## Abstract

The focus of this research is the qualitative behavior of a reaction–diffusion neural network with distributed delays and conformable derivatives under impulsive perturbations. In particular, the almost periodic behavior of the proposed model is studied using a Lyapunov-based approach. By constructing an appropriate Lyapunov-type function, criteria that guarantee the existence and uniqueness of an almost periodic state are provided. The established criteria extend a few existing results on the almost periodicity of conformable models and contribute to the development of the field. In addition, the notion of global conformable exponential stability is introduced and analyzed for the developed model. A suitable example is discussed.

## 1. Introduction

The qualitative behavior of neural networks with reaction–diffusion terms has attracted the attention of researchers in many fields of science and engineering. The main reason for the intense interest in their study lies in the possibilities of their applications in biosciences [1,2], secure communication [3], pattern formation [4], and many others [5,6,7]. In fact, the structure of a reaction–diffusion neural network model includes reaction terms as well as diffusion, which arise naturally when dealing with systems whose behavior depends on time and space. At the same time, the reaction–diffusion terms can affect the dynamic evolution of the process. This is another motivation and reason for the growing interest in their study.

Moreover, delay effects are also present in real-world reaction–diffusion systems, which has greatly enhanced the study of delayed reaction–diffusion neural network models. Numerous researchers have investigated how constant, time-varying, distributed, and infinite delays can affect the dynamics of such models [8,9,10,11].

The future development of reaction–diffusion models includes the consideration of some impulsive effects. In fact, short-term perturbations are common in the evolution of reaction–diffusion models, and this stimulates great interest in the study of impulsive reaction–diffusion neural networks [12,13,14,15]. On the one hand, impulsive effects can alter the high performance of a neural network model. Also, such effects can be used to efficiently control the desired qualitative behavior. Impulsive reaction–diffusion models are described primarily using impulsive differential equations as a modeling approach [16,17]. In addition, the theory of impulsive control [18,19] is of crucial importance in the study of their performance. Note that there are also several interesting results on the dynamics of impulsive reaction–diffusion neural networks with distributed delays [20,21]. In fact, distributed delays account for variable time delays that occur in real-world processes such as transcription and translation, which are more realistic than fixed or time-varying delays.

Another innovative approach to neural network modeling involves the use of ideas of fractional calculus. Since the dynamics in reaction–diffusion neural networks are complex, the use of fractional-order derivatives leads to more flexible models. The power ability of fractional-order neural network models to improve and generalize existing integer-order models and to increase the degree of reliability and accuracy [22,23] provokes widespread interest in the study of their characteristics. Numerous results on qualitative theory and applications of fractional-order reaction–diffusion neural network models have recently been established [24,25,26,27,28].

Part of the development of the theory of fractional calculus is related to the introduction of new derivatives of fractional order [29,30]. This is motivated by the fact that the application of classical fractional derivatives to applied models leads to some complexities in their analysis. For example, the application of the fractional Lyapunov approach requires the use of a complicated chain rule. Furthermore, some of the classical fractional derivatives do not obey Leibniz’s rule, and the corresponding Rolle’s theorem and the mean value theorem are not provided. The conformable derivative introduced in [31,32] aims to avoid the complexities in the applications of fractional-order derivatives. It is very promising in the computational aspect, as the corresponding definitions are limit-based. That is why the research on conformable calculus is growing [33,34,35,36,37]. Furthermore, the advantages of conformal derivatives expand their use in mathematical modeling [38,39,40,41]. The extensive work conducted on the analysis of applied conformable mathematical models, including conformable neural network models [42,43], shows their importance for theory and applications.

Impulsive extensions of conformable systems have also recently been investigated [44,45,46]. The hybrid impulsive conformable calculus approach is also applied to some impulsive neural network models [47,48,49,50,51]. Note that research on impulsive conformable neural networks of reaction–diffusion type is still very rare. For example, in [47], the existence and stability of integral manifolds of impulsive HCV conformable neural network models with reaction–diffusion terms are investigated. The paper [48] studies the existence and uniqueness of almost periodic solutions of conformable impulsive reaction–diffusion neural network models by constructing suitable Lyapunov-like functions. However, the authors in [47,48] did not take into account delay effects. Therefore, research on impulsive conformable reaction–diffusion neural network models needs further development.

A review of the existing literature showed that most qualitative research on conformable neural network models is concerned with stability and synchronization properties [42,43,45,47,49,50]. Progress in research on periodicity for such models is not satisfactory. The paper [44] considers a periodic boundary value problem for a class of impulsive conformable fractional integro-differential equations. However, in real-world problems, pure periodicity is not substantial. In addition, it has been shown in [52] that pure periodic solutions do not exist for fractional-order systems. Hence, the almost periodicity is an option that motivates extensive research on almost periodic solutions of fractional-order models [24,53,54,55,56]. This qualitative behavior is not fully studied for conformable neural network models. The almost periodic behavior of the solutions of impulsive conformable reaction–diffusion neural network models is studied only in [48] without considering delay factors. Very recently, the quasi-periodic behavior and chaotic nature of a conformable fractional paraxial wave equation are observed in [57]. The goal of this research is to contribute to the development of the almost periodicity for impulsive conformable neural network models considering both reaction–diffusion terms and distributed delays.

In this paper, we will define an impulsive conformable reaction–diffusion neural network model with distributed delays. The almost periodic behavior of the solutions will be analyzed using the conformable Lyapunov approach.

The main contributions of our paper can be summarized as follows:

(i) Using the hybrid impulsive conformable setting, an impulsive conformable reaction-diffusion model is constructed. Unlike [48], distributed delays are also considered. The explicit structure of the model is very general and includes some conformable models developed in the existing literature [42,43,47,49,50]. It is also very useful for overcoming a fundamental difficulty in estimating network performance from experiments, the fact that only some representative subsets of neurons can be measured simultaneously. In addition, impulsive (short-term) effects allow the application of impulsive control strategies. Entropy can also be applied to measure complexity in a neural network architecture [58];
(ii) The concept of almost periodicity is introduced to the discontinuous impulsive conformable delayed reaction–diffusion model. The notion of almost periodicity extends the concepts of periodicity applied in [44] and is well suited for conformable models;
(iii) By the construction of a suitable Lyapunov function, new criteria are provided for the existence, uniqueness, and global conformable exponential stability of almost periodic solutions.

Furthermore, a comprehensive reference is provided for researchers interested in impulsive and conformal neural network models, describing the existing literature and demonstrating the latest progress in their qualitative research.

The plan of the rest of the paper is as follows. In Section 2, some notes on conformable calculus are given. Then, the impulsive conformable reaction–diffusion neural network model with distributed delays is formulated. Definitions and lemmas related to the almost periodic notion and the Lyapunov approach are also presented. The main results on almost periodicity are established in Section 3. The global conformable exponential stability results are also presented. An example is presented in Section 4. Section 5 is the conclusion section, where a brief review of the main results of our research and their significance is presented, and extensions of our work are suggested.

## 2. Basic Theory—Model Formulation—Preliminary Notes

We consider the Euclidean *n*-dimensional space with elements $y={\left(y_{1},y_{2},\ldots ,y_{n}\right)}^{T}\in R^{n}$, and $R_{+}=\left[0,\infty \right)$.

### 2.1. Conformable Calculus Notes

We will begin with some notations and properties related to conformable derivatives. Let $\bar{r}\geq 0$ and $q\left(r\right):\left[\bar{r},\infty \right)\to R$.

**Definition** **1** ([31,32])**.** *Let 0<σ≤1. The conformable derivative of order σ with the lower limit $\bar{r}$ for the function q(r) is defined by*

$$T_{\bar{r}}^{\sigma }q\left(r\right)=\lim_{\varepsilon \to 0}\frac{q\left(r+\varepsilon {\left(r-\bar{r}\right)}^{1-\sigma }\right)-q\left(r\right)}{\varepsilon },\;r>\bar{r}.$$

Note that a function could be σ conformable differentiable at a point but not ordinary differentiable at this point [32].

Denote by

$$\begin{array}{c} B=\{\left\{r_{k}\right\}:\;r_{k}\in R,\;r_{k}<r_{k+1},\;r_{k}\neq 0,\;k\in Z,\;\lim_{k\to \pm \infty }r_{k}=\pm \infty \} \end{array}$$

the set of all unbounded and strictly increasing sequences for which a distance is defined by $\rho \left(\left\{r_{k}^{\left(1\right)}\right\},\left\{r_{k}^{\left(2\right)}\right\}\right)$, $\{r_{k}^{\left(j\right)}\}\in B$, j=1,2.

Next, following [45], for a function $q:[r_{k-1},r_{k})\to R$, the conformable derivative of order σ starting from $r_{k-1}$ is defined as

$$T_{r_{k-1}}^{\sigma }q\left(r\right)=\lim_{\varepsilon \to 0}\frac{q\left(r+\varepsilon {\left(r-r_{k-1}\right)}^{1-\sigma }\right)-q\left(r\right)}{\varepsilon },$$

and at $r_{k-1}$, we define

$$T_{r_{k-1}}^{\alpha }q\left(r_{k-1}\right)=\lim_{r\to r_{k-1}^{+}}T_{r_{k-1}}^{\sigma }q\left(r\right).$$

By $C^{\sigma }\left[\left[\bar{r},\infty \right),R\right]$ we denote the class of all functions q(r) that have *σ*- conformable derivatives for any $r\in [\bar{r},\infty )$.

**Definition** **2** ([47,48,49])**.** *The conformable integral of order 0<σ≤1 with a lower limit $\bar{r}$, of the function q(r), is*

$$I_{\bar{r}}^{\sigma }q\left(r\right)=\int_{\bar{r}}^{r}{\left(s-\bar{r}\right)}^{\sigma -1}q\left(s\right)ds.$$

The following properties of the conformable derivatives will be used in our analysis.

**Lemma** **1** ([47,48,49])**.** *For a function $q:[\bar{r},\infty )\to R$, $q\in C^{\sigma }\left[\left[\bar{r},\infty \right),R\right]$, 0<σ≤1, we have*

*(i)*  $I_{\bar{r}}^{\sigma }\left(T_{\bar{r}}^{\sigma }q\left(r\right)\right)=q\left(r\right)-q\left(\bar{r}\right),\;r>\bar{r};$
*(ii)*  *If $y\left(q\left(r\right)\right):\left[\bar{r},\infty \right)\to R$ is differentiable with respect to q(r), then for any $r\in [\bar{r},\infty )$ and q(r)≠0, we have*

$$T_{\bar{r}}^{\sigma }y\left(q\left(r\right)\right)=y^{\prime }\left(q\left(r\right)\right)T_{\bar{r}}^{\sigma }q\left(r\right),$$

*where $y^{\prime }$ is the derivative of y(·).*

Let Θ be a bounded domain in $R^{n}$ with a smooth boundary ∂Θ and a positive measure mesΘ>0 containing the origin. The time conformable derivative is defined as follows.

**Definition** **3** ([47,48,49])**.** *For a function z=z(t,y), $z:[\bar{r},\infty )\times \Theta \to R$, the limit*

$$T_{\bar{r}}^{\sigma }z\left(r,y\right)=\frac{\partial^{\sigma }z\left(r,y\right)}{\partial r^{\sigma }}|\bar{r}=\lim_{\varepsilon \to 0}\frac{z\left(r+\varepsilon {\left(r-\bar{r}\right)}^{1-\sigma },y\right)-z\left(r,y\right)}{\varepsilon },\;r>\bar{r},\;y\in \Theta$$

*is the conformable derivative along r of order σ, 0<σ≤1.*

If $\bar{r}=r_{k-1}$, k∈Z, then

$$T_{r_{k-1}}^{\sigma }z\left(r_{k-1},y\right)=\lim_{r\to r_{k-1}^{+}}T_{r_{k-1}}^{\sigma }z\left(r,y\right).$$

**Remark** **1.** *The σ conformable differentiable with respect to time functions has the same properties as the properties of the σ-conformable differentiable functions listed in Lemma 1 [45,48].*

**Definition** **4** ([31,32])**.** *The conformable exponential function $E_{\sigma }\left(\nu ,\tau \right)$ for 0<σ≤1 is defined by*

$$E_{\sigma }\left(\nu ,\tau \right)=\exp\left(\nu \frac{\tau^{\sigma }}{\sigma }\right),\;\nu \in R,\;\tau \in R_{+}.$$

**Remark** **2.** *More details on the conformable calculus can be found in [31,32,33,34,35,36,37], and for results related to impulsive conformable calculus, we refer to [44,45,46,47,48,49,50,51]. For the physical and geometrical interpretations of conformable derivatives, we refer to [36].*

### 2.2. Model Formulation

For vector functions of the type

$$z\left(r,y\right)={\left(z_{1}\left(r,y\right),z_{2}\left(r,y\right),\ldots ,z_{m}\left(r,y\right)\right)}^{T}\in R^{m},$$

throughout this paper, we will apply the norm

$$\left||z\left(r,\cdot \right)|\right|={\left[\int_{\Theta }\sum_{i=1}^{m}z_{i}^{2}\left(r,y\right)dy\right]}^{1/2}.$$

Let the functions $a_{ij}$, $b_{ij}$, and $\Omega_{i}$ be continuous in R, $D_{il}:R\times \Theta \to R_{+}$, and the constants $c_{i}>0$, i,j=1,2,…,m, l=1,2,…,n. For $\{r_{k}\}\in B$, we develop an impulsive conformable reaction–diffusion neural network model with distributed delays of the type

(1) $$\left\{\begin{array}{c} T_{r_{k-1}}^{\sigma }z_{i}\left(r,y\right)=\sum_{l=1}^{n}\frac{\partial }{\partial y_{l}}\left(D_{il}\frac{\partial z_{i}\left(r,y\right)}{\partial y_{l}}\right)-c_{i}z_{i}\left(r,y\right)+\sum_{j=1}^{m}a_{ij}\left(r\right)f_{j}\left(z_{j}\left(r,y\right)\right)\\ \;+\sum_{j=1}^{m}b_{ij}\left(r\right)\int_{-\infty }^{r}K_{ij}\left(r-s\right)f_{j}\left(z_{j}\left(s,y\right)\right)ds+\Omega_{i}\left(r\right),\;r\in \left[r_{k-1},r_{k}\right),\\ z_{i}\left(r_{k},y\right)=\gamma_{ik}\left(z_{i}\left(r_{k}^{-},y\right)\right),\;k\in Z,\;y\in \Theta , \end{array}\right.$$

where i=1,2,…,m, m≥2 corresponds to the number of neurons in the neural network model (1), $z={\left(z_{1},z_{2},\ldots ,z_{m}\right)}^{T}\in R^{m}$, $z_{i}=z_{i}\left(r,y\right)$ denotes the state of the *i*th neuronal unit at time *r* and space y∈Θ, and $f_{j}$ represents the activation functions of the *j*th unit. The functions $a_{ij}$ and $b_{ij}$ are the connection weights, the positive constant $c_{i}$ represents the rate with which the *i*th unit will reset its potential to the resting state in isolation when disconnected from the network and external input, and $\Omega_{i}$ denotes the external input for the *i*th unit. The smooth functions $D_{il}=D_{il}\left(r,y\right)\geq 0$ correspond to the diffusion operators of transmission along the *i*th unit, and $K_{ij}$ is the delay kernel, i,j=1,2,…,m, l=1,2,…,n. The elements of the sequence $\{r_{k}\}\in B$ are the impulsive moments at which abrupt changes of the nodes $z_{i}\left(r,y\right)$ from the positions $z_{i}\left(r_{k}^{-},y\right)$ to the positions $z_{i}\left(r_{k}^{+},y\right)$ are observed, $\gamma_{ik}$ represents the impulsive functions that measure the impulsive control effects on the nodes $z_{i}\left(r,y\right)$ at the instants $r_{k}$, and we have $z_{i}\left(r_{k}^{+},y\right)=z_{i}\left(r_{k},y\right)$ and $\Delta z_{i}\left(r_{k},y\right)=z_{i}\left(r_{k}^{+},y\right)-z_{i}\left(r_{k}^{-},y\right)=\gamma_{ik}\left(z_{i}\left(r_{k}^{-},y\right)\right)-z_{i}\left(r_{k}^{-},y\right)$i=1,2,…,m,k∈Z.

**Remark** **3.** *The formulated model (1) extends many existing reaction–diffusion neural network models [8,9,10,11,20,21,24,25,26,27,28] to the impulsive conformable settings considering general distributed delays. The used conformable approach allows for the flexibility in the provided framework compared with the integer-order models, and simplifications in the analysis are compared with the fractional-order models. In addition, the designed model (1) allows control signals to be applied only at fixed time intervals $r_{k}$, k∈Z. These signals with magnitudes determined by the functions $\gamma_{ik}$, i=1,2,…,m, k∈Z, can be used to impulsively control the qualitative behavior of the model.*

**Remark** **4.** *The impulsive control approach has been applied to numerous integer-order and fractional-order reaction–diffusion neural network models. For conformable models, this approach has been applied to reaction–diffusion neural networks just in [47,48] without analyzing the delay effects. Thus, our research generalizes and complements numerous important results in the existing literature. It also contributes to the development of the theory and applications of conformable neural network models.*

We denote by ${\mathrm{PC}}_{\infty }^{\Theta }$ the class of all real–valued functions φ=φ(χ,y), φ:(−∞,0]×Θ→R that are piecewise continuous with respect to χ and bounded on (−∞,0]×Θ. We also assume that the limits on the left and right sides at the points of discontinuity exist, and all functions $\varphi_{0i}\left(\chi ,y\right)$ are continuous on the right at these points. We denote the norm in ${\mathrm{PC}}_{\infty }^{\Theta }$ by

$${\left||\varphi |\right|}_{\infty }=\underset{-\infty <\chi \leq 0}{\sup}||\varphi \left(\chi ,\cdot \right)||.$$

The impulsive control conformable model (1) will be studied under boundary and initial conditions of the following type:

(2) $$z_{i}\left(r,y\right)=0,\;r\in R,\;y\in \partial \Theta ,$$

(3) $$z_{i}\left(\chi ,y\right)=\varphi_{0i}\left(\chi ,y\right),\;\chi \in \left(-\infty ,0\right],\;y\in \Theta ,$$

where $\varphi_{0}={\left(\varphi_{01},\varphi_{02},\ldots ,\varphi_{0m}\right)}^{T}$, and $\varphi_{0i}\in {\mathrm{PC}}_{\infty }^{\Theta }$ for any i=1,2,…,m.

The solution of the initial boundary value problem (IBVP) (1)–(3) will be denoted by

$$z\left(r,y\right)=z\left(r,y;\varphi_{0}\right),\;r\in R,\;y\in \Theta .$$

The function z(r,y) is piecewise continuous with respect to its first variable, with points of discontinuity of the first kind, and $z_{i}\left(r_{k}^{+},y\right)=z_{i}\left(r_{k},y\right)=\gamma_{ik}\left(z_{i}\left(r_{k}^{-},y\right)\right),$ y∈Ω, k∈Z.

### 2.3. Lyapunov Approach Definitions and Lemmas

The application of the Lyapunov analysis approach to impulsive systems requires the use of piecewise continuous Lyapunov-type functions $\Lambda :R\times R^{2m}\to R_{+}$, Λ=Λ(r,z,v) such that for $\{r_{k}\}\in B$:

(i). Λ(r,z,v) is continuous in $\left(r_{k-1},r_{k}\right)\times R^{2m}$, k∈Z and local Lipschitz continuous with respect to the arguments (z,v);
(ii). For each k∈Z and ${\left(z,v\right)}^{T}\in R^{2m}$, the finite limits
  Λ(rk−1−,z,v)=limt→rk−1t<rk−1Λ(r,z,v),Λ(rk−1+,z,v)=limt→rk−1t>rk−1Λ(r,z,v)
  exist, and $\Lambda \left(r_{k-1}^{+},z,v\right)=\Lambda \left(r_{k-1},z,v\right).$

The class of all functions Λ=Λ(r,z,v) of the above type will be denoted by $\Lambda_{r_{k-1}}^{\sigma }$.

The proof of the next Lemma is similar to the proof of Theorem 3.1 in [45], and we will omit it here.

**Lemma** **2.** *Assume that, for the piecewise continuous in R function w(r), there exist positive constants $\kappa_{1},\;\kappa_{2},\;I,\;\kappa_{1}>\kappa_{2}$, such that*

$$T_{r_{k-1}}^{\sigma }\;w\left(r\right)\leq -\kappa_{1}w\left(r\right)+\kappa_{2}\underset{-\infty <s\leq r}{\sup}w\left(s\right)+I,$$

*for $r\in [r_{k-1},r_{k}),\;k\in Z.$*

*Then,*

$$w\left(r\right)\leq \underset{s\leq r_{k-1}}{\sup}w\left(s\right)E_{\sigma }\left(-\eta_{k},r-r_{k-1}\right)+\frac{I}{\kappa_{1}-\kappa_{2}},$$

*where $\eta_{k}>0$ is determined by*

$$\kappa_{2}e^{\eta_{k}\theta_{k}}-\kappa_{1}+\eta_{k}<0,\;\theta_{k}\geq \frac{{\left(r_{k}-r_{k-1}\right)}^{\sigma }}{\sigma },$$

*for k∈Z.*

**Lemma** **3.** *Assume that for the function $\Lambda \left(r,z,v\right)\in \Lambda_{r_{k-1}}^{\sigma }$, there exist positive constants $\kappa_{1},\;\kappa_{2},\;I,\;\mu_{k},\;\kappa_{1}>\kappa_{2}$, such that*

*1*. *For* $r\in \left[r_{k-1},r_{k}\right),\;k\in Z,\;{\left(z,v\right)}^{T}\in R^{2m}$,
  $$T_{r_{k-1}}^{\sigma }\;\Lambda \left(r,z\left(r,\cdot \right),v\left(r,\cdot \right)\right)\leq -\kappa_{1}\Lambda \left(r,z\left(r,\cdot \right),v\left(r,\cdot \right)\right)+\kappa_{2}\underset{-\infty <s\leq r}{\sup}\Lambda \left(s,z\left(s,\cdot \right),v\left(s,\cdot \right)\right)+I;$$
*2*.
$\Lambda \left(r_{k},z\left(r_{k},\cdot \right),v\left(r_{k},\cdot \right)\right)\leq \mu_{k}\Lambda \left(r_{k}^{-},z\left(r_{k}^{-},\cdot \right),v\left(r_{k}^{-},\cdot \right)\right),\;k\in Z.$

*Then,*

$$\Lambda \left(r,z\left(r,\cdot \right),v\left(r,\cdot \right)\right)\leq \prod_{j=1}^{k-1}\mu_{j}\underset{-\infty <s\leq 0}{\sup}\Lambda \left(s,z\left(s,\cdot \right),v\left(s,\cdot \right)\right)E_{\sigma }\left(-\hat{\eta },r\right)+\frac{I}{\kappa_{1}-\kappa_{2}}\left[1+\sum_{o=0}^{k-2}\prod_{j=o+1}^{k-1}\mu_{j}\right],$$

*where $\hat{\eta }=\;\;\underset{k\in Z}{\sup}\eta_{k},\;r\in \left[0,\infty \right)$.*

**Proof.**  Let $r_{0}$ be the first instant in the sequence B such that $r_{0}\in \left[0,\infty \right)$. Consider the interval $\left[0,r_{0}\right)$, and from Lemma 2, we get

$$\Lambda \left(r,z\left(r,\cdot \right),v\left(r,\cdot \right)\right)\leq \underset{-\infty <s\leq 0}{\sup}\Lambda \left(s,z\left(s,\cdot \right),v\left(s,\cdot \right)\right)E_{\sigma }\left(-\eta_{1},r\right)+\frac{I}{\kappa_{1}-\kappa_{2}},$$

for any $r\in [0,r_{0})$.

Then, from condition 2 of Lemma 3, we have

$$\Lambda \left(r_{0},z\left(r_{0},\cdot \right),v\left(r_{0},\cdot \right)\right)\leq \mu_{0}\Lambda \left(r_{0}^{-},z\left(r_{0}^{-},\cdot \right),v\left(r_{0}^{-},\cdot \right)\right)$$

$$\leq \mu_{0}\left(\underset{-\infty <s\leq 0}{\sup}\Lambda \left(s,z\left(s,\cdot \right),v\left(s,\cdot \right)\right)E_{\sigma }\left(-\eta_{1},r_{0}\right)+\frac{I}{\kappa_{1}-\kappa_{2}}\right)$$

$$\leq \mu_{0}\underset{-\infty <s\leq 0}{\sup}\Lambda \left(s,z\left(s,\cdot \right),v\left(s,\cdot \right)\right)E_{\sigma }\left(-\eta_{1},r_{0}\right)+\frac{I\mu_{0}}{\kappa_{1}-\kappa_{2}}$$

Now, if $r\in [r_{0},r_{1})$, and we apply the well known inequality [45] $\zeta^{q}+\xi^{q}\geq {\left(\zeta +\xi \right)}^{q},\;\zeta ,\;\xi \geq 0,\;0<q\leq 1$, we derive the estimate

$$\Lambda \left(r,z\left(r,\cdot \right),v\left(r,\cdot \right)\right)\leq \underset{-\infty <s\leq r_{0}}{\sup}\Lambda \left(s,z\left(s,\cdot \right),v\left(s,\cdot \right)\right)E_{\sigma }\left(-\eta_{1},r-r_{0}\right)+\frac{I}{\kappa_{1}-\kappa_{2}}$$

$$\leq \left(\mu_{1}\underset{-\infty <s\leq 0}{\sup}\Lambda \left(s,z\left(s,\cdot \right),v\left(s,\cdot \right)\right)E_{\sigma }\left(-\eta_{0},r_{0}\right)+\frac{I\mu_{1}}{\kappa_{1}-\kappa_{2}}\right)E_{\sigma }\left(-\eta_{1},r-r_{0}\right)+\frac{I}{\kappa_{1}-\kappa_{2}}$$

$$\leq \mu_{1}\underset{-\infty <s\leq 0}{\sup}\Lambda \left(s,z\left(s,\cdot \right),v\left(s,\cdot \right)\right)E_{\sigma }\left(-\hat{\eta },r\right)+\frac{I\mu_{1}}{\kappa_{1}-\kappa_{2}}+\frac{I}{\kappa_{1}-\kappa_{2}}.$$

If we continue the process, then for $r\in [r_{k-1},r_{k})$, we have

$$\Lambda \left(r,z\left(r,\cdot \right),v\left(r,\cdot \right)\right)\leq \underset{-\infty <s\leq r_{k-1}}{\sup}\Lambda \left(s,z\left(s,\cdot \right),v\left(s,\cdot \right)\right)E_{\sigma }\left(-\eta_{k},r-r_{k-1}\right)+\frac{I}{\kappa_{1}-\kappa_{2}}$$

$$\leq \prod_{j=1}^{k-1}\mu_{j}\underset{-\infty <s\leq 0}{\sup}\Lambda \left(s,z\left(s,\cdot \right),v\left(s,\cdot \right)\right)E_{\alpha }\left(-\hat{\eta },r\right)+\frac{I}{\kappa_{1}-\kappa_{2}}\left[1+\sum_{o=0}^{k-2}\prod_{j=o+1}^{k-1}\mu_{j}\right].$$

□

### 2.4. Almost Periodicity Notes

The concept of almost periodicity for impulsive conformable systems will be defined in the next definitions [48].

Let $\Psi =\left(\psi \left(r,y\right),T\right)\in PC\left[R\times \Theta ,R^{m}\right]\times B$. For any infinite sequence of type ${\left\{s_{p}\right\}}_{p=1}^{\infty }$, $s_{p}\in R$, we denote by $\theta_{s_{p}}\Psi$ the sets $\left\{\psi \left(r+s_{p},y\right),T-s_{p}\right\}\subset PC\left[R\times \Theta ,R^{m}\right]\times B$ where $T-s_{p}=\left\{r_{k}-s_{p}\right\}$, k∈Z, p=1,2,….

**Definition** **5.** *A function $\psi \in PC\left[R\times \Theta ,R^{m}\right],\;\psi =\psi \left(r,y\right)$, is almost periodic piecewise continuous with respect to its first argument with jump discontinuities at the points $r_{k},\;\left\{r_{k}\right\}\in B$, if any sequence of real numbers $\left\{{s^{\prime }}_{m}\right\}$ has a subsequence $\left\{s_{p}\right\},\;s_{p}=s_{m_{p}}^{\prime }$, such that $\theta_{s_{p}}\Psi$ is compact in $PC[R\times \Theta ,R^{m}]\times B$.*

**Definition** **6.** *A sequence $\left\{\Psi_{p}\right\}$, $\Psi_{p}=\left(\psi_{p}\left(r,y\right),T_{p}\right)\in PC\left[R\times \Theta ,R^{m}\right]\times B$, converges to *Ψ* uniformly with respect to r, if the existence of an ε>0 implies the existence of a $p_{0}>0$, such that both inequalities*

$$\rho \left(T,T_{p}\right)<\varepsilon ,\;||\psi_{p}\left(r,y\right)-\psi \left(r,y\right)\left|\right|<\varepsilon$$

*hold uniformly for $p\geq p_{0}$, $r\in R\setminus \cap \{\theta_{\varepsilon }\left(s\left(T_{p}\cup T\right)\right)\},$ y∈Θ, where $s(T_{p}\cup T):B\to B$, $s(T_{p}\cup T)$ forms a strictly increasing sequence, $\theta_{\varepsilon }\left(s\left(T_{p}\cup T\right)\right)=\left\{r+\varepsilon ,\;r\in s\left(T_{p}\cup T\right)\right\}$.*

**Definition** **7.** *The set of all sequences of the type $\left\{r_{k}^{h}\right\}$, $r_{k}^{h}=r_{k+h}-r_{k}$, k,h∈Z, is uniformly almost periodic if, from each infinite sequence of shifts $\{r_{k}-s_{p}\},\;k\in Z$, $p=1,2,\ldots ,s_{p}\in R$, it is possible to derive a convergent subsequence in B.*

**Remark** **5.** *The above definitions extend the definitions on almost periodicity adopted in [48] to the reaction–diffusion case. They also generalize the almost periodic concept considered in [24,56] and allow the almost periodic behavior to be studied for solutions of conformable systems.*

The next assumptions will ensure the existence, uniqueness, and almost periodicity of the nodes [24,48]:

**Assumption** **1.** *The functions $a_{ij}\left(r\right)$, $b_{ij}\left(r\right)$ and $\Omega_{i}\left(r\right)$ are almost periodic on r, and*

$$\underset{r\in R}{\sup}|a_{ij}\left(r\right)|=a_{ij}^{M},\;\;\;\;\underset{r\in R}{\sup}\left|b_{ij}\left(r\right)\right|=b_{ij}^{M},$$

i,j=1,2,…,m.

**Assumption** **2.** *The continuous activation functions are such that*

$$|f_{i}\left(\chi_{1}\right)-f_{i}\left(\chi_{2}\right)|\leq L_{i}|\chi_{1}-\chi_{2}\left|,\;\right|f_{i}\left(\chi \right)|\leq H_{i}$$

*and $f_{i}\left(0\right)=0$ for $L_{i}>0$, $H_{i}>0$ and all $\chi_{1},\chi_{2}\in R,$ $\chi_{1}\neq \chi_{2}$, i=1,2,…,m.*

**Assumption** **3.** *The delay kernel $K_{ij}:R_{+}\to R_{+},i,j=1,2,\ldots ,m$ is a continuous function that satisfies the following conditions:*

*(i)*  *$\int_{0}^{\infty }K_{ij}\left(s\right)ds=1$;*
*(ii)*  *$\int_{0}^{\infty }K_{ij}\left(r-s\right)ds<\infty$;*
*(iii)*  $\int_{-\infty }^{r}K_{ij}\left(r-s\right)ds<\Gamma_{ij}^{*}<\infty$.

**Assumption** **4.** *The functions $D_{il}=D_{il}\left(r,y\right)$ are almost periodic in r for any y∈Θ, and*

$$D_{il}\left(r,y\right)\geq {\underline{D}}_{il}\geq 0,\;r\in R,\;y\in \Theta ,$$

i=1,2,…,m,l=1,2,…,n.

**Assumption** **5.** *For any i=1,2,…,m and k∈Z, the impulsive functions $\gamma_{ik}$ are $\gamma_{ik}\left(z_{i}\left(r_{k}^{-},y\right)\right)=H_{ik}z_{i}\left(r_{k}^{-},y\right)$, $H_{ik}\in R$, y∈Θ, and*

$$H_{ik}^{2}\leq \mu_{k},$$

*where $\mu_{k}>0$, i=1,2,…,m,k∈Z.*

**Assumption** **6.** *The set of all sequences $\left\{r_{k}^{h}\right\}$, $r_{k}^{h}=r_{k+h}-r_{k},\;k,h\in Z$ is uniformly almost periodic, and $\inf_{k}r_{k}^{1}=\tau >0$.*

**Assumption** **7.** *The initial function $\varphi_{0}={\left(\varphi_{01},\varphi_{02},\ldots ,\varphi_{0m}\right)}^{T}$, $\varphi_{0i}=\varphi_{0i}\left(\chi ,y\right)$, $\varphi_{0i}\in {\mathrm{PC}}_{\infty }^{\Theta }$, i=1,2,…,m, is almost periodic with respect to its first argument.*

It follows that [24,48], under Assumptions 1–7, for an arbitrary infinite sequence of real numbers $\left\{s_{q}^{\prime }\right\}$, there exists a subsequence $\left\{s_{p}\right\},\;s_{p}=s_{q_{p}}^{\prime }$ that shifts system (1) to the system

(4) $$\left\{\begin{array}{c} T_{r_{k-1}}^{\sigma }z_{i}\left(r,y\right)=\sum_{l=1}^{n}\frac{\partial }{\partial y_{l}}\left(D_{il}\frac{\partial z_{i}\left(r,y\right)}{\partial y_{l}}\right)-c_{i}z_{i}\left(r,y\right)+\sum_{j=1}^{m}a_{ij}^{s}\left(r\right)f_{j}\left(z_{j}\left(r,y\right)\right)\\ \;+\sum_{j=1}^{m}b_{ij}^{s}\left(r\right)\int_{-\infty }^{r}K_{ij}\left(r-s\right)f_{j}\left(z_{j}\left(s,y\right)\right)ds+\Omega_{i}^{s}\left(r\right),\;r\neq r_{k}^{s},\\ z_{i}\left(r_{k}^{s},y\right)=\gamma_{ik}(z_{i}\left(r_{k}^{s-},y)\right),\;k\in Z,\;y\in \Theta . \end{array}\right.$$

The set of all displaced systems of type (4) will be denoted by $H(z,r_{k})$.

## 3. Main Results

### 3.1. A Lyapunov-Based Analysis on the Almost Periodicity

We will consider the impulsive conformable model (1) on R×Θ with

$$\Theta =\{y={\left(y_{1},y_{2},\ldots ,y_{n}\right)}^{T}\in R^{n},\;|y_{l}|<\nu_{l}\},$$

where $\nu_{l}$(l=1,2,…,n) are positive constants.

The following lemma will be useful in the proof of the main result.

**Lemma** **4** ([9])**.** *For a real–valued function u(y), $u\in C^{1}\left(\Theta \right)$ such that ${u\left(y\right)|}_{\Theta }=0$, we have*

$$\int_{\Theta }u^{2}\left(y\right)dy\leq \nu_{l}^{2}\int_{\Theta }|\frac{\partial u(y)}{\partial y_{l}}{|}^{2}dy.$$

**Theorem** **1.** *Assume that Assumptions 1–7 are satisfied, and*

*1*. *There exist positive numbers $\kappa_{1}$, $\kappa_{2}$, $d_{i}$, such that*
$$\kappa_{1}=\min_{1\leq i\leq m}\left(2\left(c_{i}+d_{i}\right)-\sum_{j=1}^{m}\left(a_{ij}^{M}+b_{ij}^{M}\Gamma_{ij}^{*}\right)L_{j}\right)>0,$$
$$\kappa_{2}=\max_{1\leq i\leq m}\sum_{j=1}^{m}b_{ij}^{M}L_{j}\Gamma_{ij}^{*}>0,$$
$$d_{i}=\sum_{l=1}^{n}\frac{{\underline{D}}_{il}}{\nu_{l}^{2}},\;i=1,2,\ldots ,m,$$
  *and $\kappa_{1}>\kappa_{2}$.*
*2*. *There is a solution z(r,y) of (1), such that*
||z(r,y)||<C,C>0,
  *for r≥0 and y∈Θ.*

*Then, there exists a unique almost periodic along r solution β(r,y) of system (1), uniformly on y∈Θ, such that*

*(a)*  *$\left||\beta \left(r,y\right)|\right|\leq C_{1},\;C_{1}<C$;*
*(b)*  *$H\left(\beta ,r_{k}\right)\subset H\left(z,r_{k}\right)$.*

**Proof.**  Consider an infinite sequence of real numbers $\left\{s_{p}\right\}$ that moves system (1) to a system from the set $H(z,r_{k})$, such that $s_{p}\to \infty$ as p→∞.

Let z(r,y) and v(r,y) be two solutions to system (1), (r,y)∈R×Θ. We define a Lyapunov function

$$\Lambda \left(z\left(r,\cdot \right),v\left(r,\cdot \right)\right)=\int_{\Theta }\frac{1}{2}\sum_{i=1}^{m}{\left(z_{i}\left(r,y\right)-v_{i}\left(r,y\right)\right)}^{2}dy.$$

For any real number *b*, let $p_{0}=p_{0}\left(b\right)$ be the smallest value of *p*, such that $s_{p_{0}}+b\geq 0$. Condition 2 of Theorem 1 implies the existence of a $C_{1}<C$, such that ${\left||z\left(r,y\right)|\right|}_{2}\leq C_{1}$ for all t≥0, and hence, $||z\left(r+s_{p},y\right){\left|\right|}_{2}\leq C_{1}$ for $r\geq b,\;p\geq p_{0}$, y∈Θ.

Suppose that Π,Π⊂(b,∞) is compact. Hence, for every ε>0, we can pick out an integer number $n_{0}\left(\varepsilon ,b\right)\geq p_{0}\left(b\right)$, so large that, for $h\geq p\geq n_{0}\left(\varepsilon ,b\right)$ and $r\in R,\;r\neq r_{k},\;k\in Z$, we have

(5) $$|\Omega_{i}\left(r+s_{p}\right)-\Omega_{i}\left(r+s_{h}\right)|<\varepsilon^{2}\left(\kappa_{1}-\kappa_{2}\right){\left(24mC{\left(\nu_{l}\right)}^{n}\left[1+\sum_{j=0}^{k-2}\prod_{i=j+1}^{k-1}\mu_{i}\right]\right)}^{-1},$$

(6) $$|a_{ij}\left(r+s_{p}\right)-a_{ij}\left(r+s_{h}\right)|\leq \varepsilon^{2}\left(\kappa_{1}-\kappa_{2}\right){\left(24mC2^{n}{\left(\nu_{l}\right)}^{n}\left[1+\sum_{j=0}^{k-2}\prod_{i=j+1}^{k-1}\mu_{i}\right]\sum_{j=1}^{m}H_{j}\right)}^{-1},$$

(7) $$|b_{ij}\left(r+s_{p}\right)-b_{ij}\left(r+s_{h}\right)|\leq \varepsilon^{2}\left(\kappa_{1}-\kappa_{2}\right){\left(24mC2^{n}{\left(\nu_{l}\right)}^{n}\left[1+\sum_{j=0}^{k-2}\prod_{i=j+1}^{k-1}\mu_{i}\right]\sum_{j=1}^{m}H_{j}\Gamma^{*}\right)}^{-1},$$

and

(8) $$\prod_{i=1}^{k-1}\mu_{i}\left[\underset{-\infty <s\leq 0}{\sup}\int_{\Theta }\sum_{i=1}^{m}{\left(\varphi_{0i}\left(s+s_{p},y\right)-\varphi_{0i}\left(s+s_{h},y\right)\right)}^{2}dy\right]E_{\sigma }\left(-\hat{\eta },r+s_{p}-s_{h}\right)<\frac{\varepsilon^{2}}{2}.$$

Then, by Assumption 5, for $r=r_{k}$, we obtain

(9) $$\begin{array}{cc} \Lambda (z\left(r_{k}^{+}+s_{p},\cdot \right),z\left(r_{k}^{+}+s_{h},\cdot \right)) & =\int_{\Theta }\frac{1}{2}\sum_{i=1}^{m}(H_{ik}{\left(z_{i}\left(r_{k}^{-}+s_{p},y\right)-z_{i}\left(r_{k}^{-}+s_{h},y\right)\right)}^{2}dy\\ \; & \leq \mu_{k}\int_{\Theta }\frac{1}{2}\sum_{i=1}^{m}{\left(z_{i}\left(r_{k}^{-}+s_{p},y\right)-z_{i}\left(r_{k}^{-}+s_{h},y\right)\right)}^{2}dy\\ \; & =\mu_{k}\Lambda \left(z\left(r_{k}^{-}+s_{p},\cdot \right),z\left(r_{k}^{-}+s_{h},\cdot \right)\right). \end{array}$$

Now, we will consider the derivative $T_{r_{k-1}}^{\sigma }\;\Lambda \left(r,z\left(r,\cdot \right),v\left(r,\cdot \right)\right)$ of the function Λ for $r\in \left[r_{k-1},r_{k}\right),\;k\in Z,\;{\left(z,v\right)}^{T}\in R^{2m}$. We have that [48]

(10) $$\begin{array}{cc} T_{r_{k-1}}^{\sigma }\;\Lambda \left(z\left(r,\cdot \right),v\left(r,\cdot \right)\right) & =\frac{1}{2}T_{r_{k-1}}^{\sigma }\;\left(\int_{\Theta }\sum_{i=1}^{m}{\left(z_{i}\left(r,y\right)-v_{i}\left(r,y\right)\right)}^{2}dy\right)\\ \; & =\frac{1}{2}\sum_{i=1}^{m}\left(\int_{\Theta }T_{r_{k-1}}^{\sigma }{\left(z_{i}\left(r,y\right)-v_{i}\left(r,y\right)\right)}^{2}dy\right)\\ \; & =\sum_{i=1}^{m}\left(\int_{\Theta }\left(z_{i}\left(r,y\right)-v_{i}\left(r,y\right)\right)T_{r_{k-1}}^{\sigma }\left(z_{i}\left(r,y\right)-v_{i}\left(r,y\right)\right)dy\right). \end{array}$$

Hence, along with the system (1), we derive

$$T_{r_{k-1}}^{\sigma }\;\Lambda \left(z\left(r+s_{p},\cdot \right),z\left(r+s_{h},\cdot \right)\right)$$

$$=\sum_{i=1}^{m}\int_{\Theta }\left(z_{i}\left(r+s_{p},y\right)-z_{i}\left(r+s_{h},y\right)\right)(\sum_{l=1}^{n}\frac{\partial }{\partial y_{l}}\left(D_{il}\frac{\partial }{\partial y_{l}}\left(z_{i}\left(r+s_{p},y\right)-z_{i}\left(r+s_{h},y\right)\right)\right)$$

$$-c_{i}\left(z_{i}\left(r+s_{p},y\right)-z_{i}\left(r+s_{h},y\right)\right)+\sum_{j=1}^{m}(\left(a_{ij}\left(r+s_{p}\right)-a_{ij}\left(r+s_{h}\right)\right)f_{j}\left(z_{j}\left(r+s_{p},y\right)\right)$$

$$+a_{ij}\left(r+s_{h}\right)\left(f_{j}\left(z_{j}\left(r+s_{p},y\right)\right)-f_{j}\left(z_{j}\left(r+s_{h},y\right)\right)\right))$$

$$+\sum_{j=1}^{m}(\left(b_{ij}\left(r+s_{p}\right)-b_{ij}\left(r+s_{h}\right)\right)\int_{-\infty }^{r}K_{ij}\left(r-s\right)f_{j}(z_{j}\left(s+s_{p},y\right)ds$$

$$+\sum_{j=1}^{m}b_{ij}\left(r+s_{h}\right)(\int_{-\infty }^{r}K_{ij}\left(r-s\right)f_{j}\left(z_{j}\left(s+s_{p},y\right)\right)ds-\int_{-\infty }^{r}K_{ij}\left(r-s\right)f_{j}\left(z_{j}\left(s+s_{h},y\right))ds\right)$$

$$+\Omega_{i}\left(r+s_{p}\right)-\Omega_{i}\left(r+s_{h}\right))dy.$$

We introduce the notation $\varpi_{i}\left(r,y\right)=z_{i}\left(r+s_{p},y\right)-z_{i}\left(r+s_{h},y\right)$. Then, by the boundary conditions and the Green’s identity, we have

$$\sum_{l=1}^{n}\int_{\Theta }\varpi_{i}\left(r,y\right)\frac{\partial }{\partial y_{l}}\left(D_{il}\frac{\partial \varpi_{i}\left(r,y\right)}{\partial y_{l}}\right)dy=-\sum_{l=1}^{n}\int_{\Theta }D_{il}{\left(\frac{\partial \varpi_{i}\left(r,y\right)}{\partial y_{l}}\right)}^{2}dy.$$

We apply to the above equality, Assumption 4, condition 1 of Theorem 1, and Lemma 4 and obtain

(11) $$\begin{array}{c} \sum_{l=1}^{n}\int_{\Theta }\varpi_{i}\left(r,y\right)\frac{\partial }{\partial y_{l}}\left(D_{il}\frac{\partial \varpi_{i}\left(r,y\right)}{\partial y_{l}}\right)dy\leq -\sum_{l=1}^{n}\int_{\Theta }{\underline{D}}_{il}{\left(\frac{\partial \varpi_{i}\left(r,y\right)}{\partial y_{l}}\right)}^{2}dy\\ \leq -\sum_{l=1}^{n}\int_{\Theta }\frac{{\underline{D}}_{il}}{\nu_{l}^{2}}\varpi_{i}^{2}\left(r,y\right)dy=-d_{i}\int_{\Theta }\varpi_{i}^{2}\left(r,y\right)dy. \end{array}$$

Next, by Assumption 1, Assumption 2, and (6), we get

(12) $$\begin{array}{c} \sum_{j=1}^{m}(\left(a_{ij}\left(r+s_{p}\right)-a_{ij}\left(r+s_{h}\right)\right)\int_{\Theta }\varpi_{i}\left(r,y\right)f_{j}\left(z_{j}\left(r+s_{p},y\right)\right)dy\\ +a_{ij}\left(r+s_{q}\right)\int_{\Theta }\varpi_{i}\left(r,y\right)\left(f_{j}\left(z_{j}\left(r+s_{p},y\right)\right)-f_{j}\left(z_{j}\left(r+s_{h},y\right)\right)\right)dy)\\ \leq \frac{\varepsilon^{2}\left(\kappa_{1}-\kappa_{2}\right)}{12m}{\left[1+\sum_{j=0}^{k-2}\prod_{i=j+1}^{k-1}\mu_{i}\right]}^{-1}+\sum_{j=1}^{m}a_{ij}^{M}L_{j}\int_{\Theta }|\varpi_{i}\left(r,y\right)||\varpi_{j}\left(r,y\right)|dy\\ \leq \frac{\varepsilon^{2}\left(\kappa_{1}-\kappa_{2}\right)}{12m}{\left[1+\sum_{j=0}^{k-2}\prod_{i=j+1}^{k-1}\mu_{i}\right]}^{-1}+\frac{1}{2}\sum_{j=1}^{m}a_{ij}^{M}L_{j}\int_{\Theta }\left(\varpi_{i}^{2}\left(r,y\right)+\varpi_{j}^{2}\left(r,y\right)\right)dy. \end{array}$$

Analogously, from Assumptions 1–3 and (7), we obtain

(13) $$\begin{array}{c} \sum_{j=1}^{m}(\left(b_{ij}\left(r+s_{p}\right)-b_{ij}\left(r+s_{h}\right)\right)\int_{\Theta }\varpi_{i}\left(r,y\right)\left(\int_{-\infty }^{r}K_{ij}\left(r-s\right)f_{j}\left(z_{j}\left(s+s_{p},y\right)\right)ds\right)dy\\ +\sum_{j=1}^{m}b_{ij}\left(r+s_{h}\right)\int_{\Theta }\varpi_{i}\left(r,y\right)(\int_{-\infty }^{r}K_{ij}\left(r-s\right)\left(f_{j}\left(z_{j}\left(s+s_{p},y\right)\right)-f_{j}\left(z_{j}\left(s+s_{h},y\right))\right)ds\right)dy\\ \leq \frac{\varepsilon^{2}\left(\kappa_{1}-\kappa_{2}\right)}{12m}{\left[1+\sum_{j=0}^{k-2}\prod_{i=j+1}^{k-1}\mu_{i}\right]}^{-1}\\ +\sum_{j=1}^{m}b_{ij}^{M}|L_{j}\int_{-\infty }^{r}K_{ij}\left(r-s\right)\left(\int_{\Theta }|\varpi_{i}\left(r,y\right)||\varpi_{j}\left(s,y\right)|dy\right)ds\\ \leq \frac{\varepsilon^{2}\left(\kappa_{1}-\kappa_{2}\right)}{12m}{\left[1+\sum_{j=0}^{k-2}\prod_{i=j+1}^{k-1}\mu_{i}\right]}^{-1}+\sum_{j=1}^{m}b_{ij}^{M}L_{j}\Gamma_{ij}^{*}\int_{\Theta }\varpi_{i}^{2}\left(r,y\right))dy\\ +\sum_{j=1}^{m}b_{ij}^{M}L_{j}\Gamma_{ij}^{*}\underset{-\infty <s\leq r}{\sup}\int_{\Theta }\varpi_{j}^{2}\left(s,y\right)dy. \end{array}$$

Then, by (10)–(13) and condition 1 of Theorem 1, we get

$$T_{r_{k-1}}^{\sigma }\;\Lambda \left(z\left(r+s_{p},\cdot \right),z\left(r+s_{h},\cdot \right)\right)$$

$$\leq -\kappa_{1}\Lambda \left(z\left(r+s_{p},\cdot \right),z\left(r+s_{h},\cdot \right)\right)+\kappa_{2}\underset{-\infty <s\leq r}{\sup}\Lambda \left(z\left(s+s_{p},\cdot \right),z\left(s+s_{h},\cdot \right)\right)$$

$$+\varepsilon^{2}\left(\kappa_{1}-\kappa_{2}\right){\left(4\left[1+\sum_{j=0}^{k-2}\prod_{i=j+1}^{k-1}\mu_{i}\right]\right)}^{-1}.$$

Now, from the last estimate, (9), and Lemma 3, we have

$$\Lambda (z\left(r+s_{p},\cdot \right),z\left(r+s_{h},\cdot \right))$$

$$\leq \prod_{i=1}^{k-1}\mu_{i}\underset{-\infty <s\leq 0}{\sup}\Lambda \left(z\left(s,\cdot \right),z\left(s,\cdot \right)\right)E_{\sigma }\left(-\hat{\eta },r+s_{p}-s_{h}\right)+\frac{\varepsilon^{2}}{4},$$

or from the definition of the function Λ, it follows that

$$\int_{\Theta }\frac{1}{2}\sum_{i=1}^{m}{\left(z_{i}\left(r+s_{p},y\right)-z_{i}\left(r+s_{h},y\right)\right)}^{2}dy$$

$$\leq \prod_{i=1}^{k-1}\mu_{i}\left[\underset{-\infty <s\leq 0}{\sup}\int_{\Theta }\frac{1}{2}\sum_{i=1}^{m}{\left(\varphi_{0i}\left(s+s_{p},y\right)-\varphi_{0i}\left(s+s_{h},y\right)\right)}^{2}dy\right]E_{\sigma }\left(-\hat{\eta },r+s_{p}-s_{h}\right)+\frac{\varepsilon^{2}}{4}.$$

Then,

(14) $$\begin{array}{c} ||z\left(r+s_{p},y\right)-z\left(r+s_{h},y\right){\left|\right|}^{2}\leq \prod_{i=1}^{k-1}\mu_{i}[\underset{-\infty <s\leq 0}{\sup}\int_{\Theta }\sum_{i=1}^{m}(\varphi_{0i}\left(s+s_{p},y\right)\\ -\varphi_{0i}\left(s+s_{h},y\right){)}^{2}dy]E_{\sigma }\left(-\hat{\eta },r+s_{p}-s_{h}\right)+\frac{\varepsilon^{2}}{2}. \end{array}$$

Next, from (14) and (8), we obtain

(15) $$||z\left(r+s_{p},y\right)-z\left(r+s_{h},y\right)\left|\right|<{\left(\frac{\varepsilon^{2}}{2}+\frac{\varepsilon^{2}}{2}\right)}^{\frac{1}{2}}=\varepsilon .$$

Therefore, there exists a function $\beta \left(r,y\right)={\left(\beta_{1}\left(r,y\right),\beta_{2}\left(r,y\right),\ldots ,\beta_{m}\left(r,y\right)\right)}^{T}$ for which $z\left(r+s_{p},y\right)\to \beta \left(r,y\right)$ as p→∞ uniformly on y∈Θ. The arbitrariness of the real number *b* implies that β(r,y) is also defined uniformly on r∈R.

In a similar way, for any ε, we can prove the next inequality

$$|T_{r_{k-1}}^{\sigma }z_{i}\left(r+s_{p},y\right)-T_{r_{k-1}}^{\sigma }z_{i}\left(r+s_{h},y\right)|<\varepsilon ,$$

holds, or $\lim_{p\to \infty }T_{r_{k-1}}^{\sigma }z_{i}\left(r+s_{p},y\right)$ exists uniformly on all compact subsets of $R,r\neq r_{k}$, k∈Z, y∈Θ.

Then,

(16) $$T_{r_{k-1}}^{\sigma }\beta_{i}\left(r,y\right)=F_{i}^{s}\left(r,\beta_{i}\left(r,y\right)\right),\;r\neq r_{k}^{s},$$

where $r_{k}^{s}=\lim_{p\to \infty }\left(r_{k}+s_{p}\right)$, y∈Θ, and

(17) $$\begin{array}{c} F_{i}^{s}\left(r,\beta_{i}\left(r,y\right)\right)=\sum_{l=1}^{n}\frac{\partial }{\partial y_{l}}\left(D_{il}\frac{\partial \beta_{i}\left(r,y\right)}{\partial y_{l}}\right)-c_{i}\beta_{i}\left(r,y\right)+\sum_{j=1}^{m}a_{ij}^{s}\left(r\right)f_{j}\left(\beta_{j}\left(r,y\right)\right)\\ +\sum_{j=1}^{m}b_{ij}^{s}\left(r\right)\int_{-\infty }^{r}K_{ij}\left(r-s\right)f_{j}\left(\beta_{j}\left(s,y\right)ds\right)+\Omega_{i}^{s}\left(r\right),\;i=1,2,\ldots ,m. \end{array}$$

In addition, for $r=r_{k}^{s}$ we get

(18) $$\beta_{i}\left(r_{k}^{s},y\right)=\lim_{p\to \infty }z_{i}\left(r_{k}^{s}+s_{p},y\right)=\lim_{p\to \infty }\gamma_{ik}z_{i}\left(r_{k}^{s-}+s_{p},y\right)=\gamma_{ik}\beta_{i}\left(r_{k}^{s-},y\right),\;i=1,2,\ldots ,m.$$

Hence, (17) and (18) imply that β(r,y) is a solution of (4).

To prove the almost periodicity of the solution β(r,y), we will use the sequence $\left\{s_{p}\right\}$ that moves the system (1) to $H(z,r_{k})$.

If we consider the function

$$\Lambda_{1}\left(\beta \left(r,\cdot \right),\omega \left(r+s_{p}-s_{h},\cdot \right)\right)=\int_{\Theta }\frac{1}{2}\sum_{i=1}^{m}{\left(\beta_{i}\left(r,y\right)-\beta_{i}\left(r+s_{p}-s_{h},y\right)\right)}^{2}dy,$$

then using the same arguments as for the function Λ, for $\bar{r}\neq r_{k-1}$, we have

(19) $$\begin{array}{c} T_{r_{k-1}}^{\sigma }\;\Lambda_{1}\left(\beta \left(r,\cdot \right),\beta \left(r+s_{p}-s_{h},\cdot \right)\right)\\ \leq -\kappa_{1}\Lambda_{1}\left(\beta \left(r,\cdot \right),\beta \left(r+s_{p}-s_{h},\cdot \right)\right)+\kappa_{2}\underset{-\infty <s\leq r}{\sup}\Lambda_{1}\left(\beta \left(s,\cdot \right),\beta \left(s+s_{p}-s_{h},\cdot \right)\right)\\ +\varepsilon^{2}\left(\kappa_{1}-\kappa_{2}\right){\left(4\left[1+\sum_{j=0}^{k-2}\prod_{i=j+1}^{k-1}\mu_{i}\right]\right)}^{-1}, \end{array}$$

and

(20) $$\Lambda_{1}\left(\beta \left(r_{k}^{s},\cdot \right),\beta \left(r_{k}^{s}+s_{p}-s_{h},\cdot \right)\right)\leq \mu_{k}\Lambda_{1}\left(\beta \left(r_{k}^{s-},\cdot \right),\beta \left(r_{k}^{s}+s_{p}-s_{h},\cdot \right)\right),\;k\in Z.$$

Hence, from (19), (20), and Lemma 3, we have

(21) $$||\beta \left(r+s_{p},y\right)-\beta \left(r+s_{h},y\right)\left|\right|<\varepsilon ,\;h\geq p\geq p_{0}\left(\varepsilon \right).$$

Finally, since the sequence $\left\{s_{p}\right\}$ is such that $\rho (r_{k}+s_{p},r_{k}+s_{h})<\varepsilon$ for $h\geq p\geq p_{0}\left(\varepsilon \right)$, $\beta (r+s_{p},y)$ converges uniformly to β(r,y).

Hence, we immediately obtain the results in statements (a) and (b) of Theorem 1, and the proof is complete. □

**Remark** **6.** *The almost periodic behavior of fractional-order neural networks is investigated by numerous researchers, and interesting results have been published in [53,54,55,56]. Reaction diffusion terms have been considered in [24]. Theorem 1 extends all these results to the confirmable setting. Thus, our results complement some results in the existing literature and contribute to the development of theory. In addition, since the application of conformal calculus avoids the complexities of analyzing fractional-order systems, the proposed results are well-suited for applied models.*

**Remark** **7.** *An analysis of the almost periodicity for impulsive conformable reaction–diffusion neural network models has been made just in [48] ignoring delay factors. Thus, compared with [48], distributed delays are taken into account in the impulsive conformable neural network (1); that is, the model studied in [48] is a special case of (1). The consideration of distributed delays makes the developed model more complex and challenging, and the qualitative results proposed are more general.*

**Remark** **8.** *The Lipschitz conditions for the activation functions in A2 are essential in the proof of our main almost periodicity results. In scenarios with strongly nonlinear activation functions, modified criteria that allow the introduced framework to deal with such nonlinearities without losing result rigor may be required. This is an interesting open problem for future investigations.*

### 3.2. Stability Analysis

Since the stability is the most investigated qualitative behavior for numerous applied models, we will study the global conformable exponential stability of the almost periodic solution of (1). To this end, we will adopt the following definition [59].

**Definition** **8.** *A solution $z\left(r,y\right)=z\left(r,y;\varphi_{0}\right),\;r\in R,\;y\in \Theta$ of model (1), corresponding to an initial function $\varphi_{0}={\left(\varphi_{01},\varphi_{02},\ldots ,\varphi_{0m}\right)}^{T}$, $\varphi_{0i}\in {\mathrm{PC}}_{\infty }^{\Theta }$, i=1,2,…,m, is globally conformably exponentially stable if there exist positive constants M and κ such that, for any other solution $v\left(r,y\right)=v\left(r,y;{\tilde{\varphi }}_{0}\right),\;r\in R,\;y\in \Theta$ of model (1), corresponding to an initial function ${\tilde{\varphi }}_{0}={\left({\tilde{\varphi }}_{01},{\tilde{\varphi }}_{02},\ldots ,{\tilde{\varphi }}_{0m}\right)}^{T}$, ${\tilde{\varphi }}_{0i}\in {\mathrm{PC}}_{\infty }^{\Theta }$, i=1,2,…,m, we have*

$$||z\left(r,y\right)-v\left(r,y\right)||\leq M||\varphi_{0}-{\tilde{\varphi }}_{0}{\left|\right|}_{\infty }E_{\sigma }\left(-\kappa ,r\right)\;t\geq 0.$$

**Remark** **9.** *It is well known that, for integer-order neural network models, the global exponential stability is a very important behavior as it guarantees fast convergence [9,14]. The fractional-order analogue is the Mittag–Leffler stability [27]. With Definition 8, we extend the global exponential stability notion to the conformable case. Similar definitions are given in [31,45].*

**Theorem** **2.** *Under the conditions of Theorem 1, the unique almost periodic solution β(r,y) of system (1) is globally conformably exponentially stable.*

**Proof.**  We denote by

$$\hat{\beta }\left(r,y\right)=\bar{\beta }\left(r,y\right)-\beta \left(r,y\right),$$

$$F^{s}\left(r,\hat{\beta }\left(r,y\right)\right)=F^{s}\left(r,\hat{\beta }\left(r,y\right)+\beta \left(r,y\right)\right)-F^{s}\left(r,\beta \left(r,y\right)\right),$$

where $\bar{\beta }\left(r,y\right)$ is any solution of (4), $F^{s}={\left(F_{1}^{s},F_{2}^{s},\ldots ,F_{m}^{s}\right)}^{T}$.

Now, we consider the system

(22) $$\left\{\begin{array}{c} T_{r_{k-1}}^{\sigma }\hat{\beta }\left(r,y\right)=F^{s}(r,\hat{\beta }\left(r,y\right),\;r\in \left[r_{k-1},r_{k}\right),\\ \hat{\beta }\left(r_{k},y\right)=\mu_{k}\hat{\beta }\left(r_{k}^{-},y\right),\;k\in Z,\;y\in \Theta , \end{array}\right.$$

and the Lyapunov function $\Lambda (\beta \left(r,\cdot \right),\beta \left(r,\cdot \right)+\hat{\beta }\left(r,\cdot \right))$.

Applying Lemma 3, we conclude that the zero solution $\hat{\beta }\left(r,y\right)=0$, (r,y)∈R×Θ of (22) is globally conformably exponentially stable, which implies the global conformable exponential stability of the almost periodic solution β(r,y) of (1). □

**Remark** **10.** *Although the stability of impulsive conformable neural networks is studied in articles [47,49], the results obtained there are not applicable to model (1), since the impulsive control functions $\gamma_{ik}$ are restricted to satisfy $|H_{ik}|<1$, i=1,2,…,m, k∈Z. The application of the new Lemma 3 allows for the use of more general impulsive functions that satisfy Assumption 5, making the proposed results less restrictive.*

**Remark** **11.** *In most of the impulsive control approaches applied to conformable systems the authors constrained the upper bounds of the impulsive intervals and on the distances between the impulsive moments [59,60]. One advantage of the proposed stability criteria is that they do not include restrictions on the distance between the impulsive control instances. Hence, the proposed technique improves some recently proposed impulsive control strategies to conformable models in [59,60].*

**Remark** **12.** *The established stability results can be successfully applied in synchronization and control problems. Additionally, they can be extended to other stability concepts such as practical exponential stability as in [61], perfect exponential stability as in [24], or predefined time stability as in [62,63]. Thus, our results open the door to future contributions on the topic.*

## 4. An Example

In this example, we consider the impulsive conformable delayed reaction–diffusion neural network model (1) for n=m=2 on the set $\Theta \subset R^{2}$, $\Theta =\{y={\left(y_{1},y_{2}\right)}^{T}\in R^{2},\;|y_{l}|<1\}$, l=1,2, given by

(23) $$\left\{\begin{array}{c} T_{r_{k-1}}^{\sigma }z_{i}\left(r,y\right)=\sum_{l=1}^{n}\frac{\partial }{\partial y_{l}}\left(D_{il}\frac{\partial z_{i}\left(r,y\right)}{\partial y_{l}}\right)-c_{i}z_{i}\left(r,y\right)+\sum_{j=1}^{m}a_{ij}\left(r\right)f_{j}\left(z_{j}\left(r,y\right)\right)\\ \;+\sum_{j=1}^{m}b_{ij}\left(r\right)\int_{-\infty }^{r}K_{ij}\left(r-s\right)f_{j}\left(z_{j}\left(s,y\right)ds\right)+\Omega_{i}\left(r\right),\;r\in \left[r_{k-1},r_{k}\right),\\ z_{i}\left(r_{k},y\right)=\gamma_{ik}\left(z_{i}\left(r_{k}^{-},y\right)\right)=\left(\begin{array}{cc} 0.04635 & 0\\ 0 & 0.00168 \end{array}\right)\\ \;+\left(\begin{array}{cc} 0.7 & 0\\ 0 & 0.9 \end{array}\right)z_{i}\left(r_{k}^{-},y\right),\;k\in Z,\;y\in \Theta , \end{array}\right.$$

where r∈R, σ=0.9, i=1,2, $r_{k}\in B$, $r_{k}$ satisfy A6, $\Omega_{1}=0.040002$, $\Omega_{2}=0.058395$, $f_{i}\left(z_{i}\right)=\frac{1}{2}\left(\right|z_{i}+1|-|z_{i}-1\left|\right)$, $K_{ij}\left(s\right)=e^{-s}$, $c_{1}=0.2$, $c_{2}=0.3$,

$${\left(a_{ij}\right)}_{2\times 2}\left(r\right)=\left(\begin{array}{cc} a_{11}\left(r\right) & a_{12}\left(r\right)\\ a_{21}\left(r\right) & a_{22}\left(r\right) \end{array}\right)=\left(\begin{array}{cc} -0.03-\sin(r\sqrt{2}) & 0.02-\cos(r\sqrt{3})\\ 0.04+\cos(r\sqrt{2}) & 0.01-\sin(r\sqrt{3}) \end{array}\right),$$

$${\left(b_{ij}\right)}_{2\times 2}\left(r\right)=\left(\begin{array}{cc} b_{11}\left(r\right) & b_{12}\left(r\right)\\ b_{21}\left(r\right) & b_{22}\left(r\right) \end{array}\right)=\left(\begin{array}{cc} -0.03+\sin(r\sqrt{2}) & -0.01+\cos(r\sqrt{3})\\ 0.01-\cos(r\sqrt{2}) & -0.01+\sin(r\sqrt{3}) \end{array}\right),$$

$${\left(D_{iq}\right)}_{2\times 2}=\left(\begin{array}{cc} 4+\sin r\sqrt{2} & 0\\ 0 & 5+\cos r\sqrt{3} \end{array}\right).$$

Obviously, for model (23), all Assumptions 1–5 are satisfied for

$${\left(a_{ij}^{M}\right)}_{2\times 2}=\left(\begin{array}{cc} 1.03 & 1.02\\ 1.04 & 1.01 \end{array}\right),\;{\left(b_{ij}^{M}\right)}_{2\times 2}=\left(\begin{array}{cc} 1.03 & 1.01\\ 1.01 & 1.01 \end{array}\right),$$

$L_{j}=1$, $\Gamma_{ij}^{*}=1$, i,j=1,2, $\mu_{k}\geq 1$, k∈Z.

Condition 1 of Theorem 1 holds for $d_{1}=3$, $d_{2}=4$,

$$\kappa_{1}=\min_{1\leq i\leq m}\left(2\left(c_{i}+d_{i}\right)-\sum_{j=1}^{m}\left(a_{ij}^{M}+b_{ij}^{M}\Gamma_{ij}^{*}\right)L_{j}\right)=2.31>0,$$

$$\kappa_{2}=\max_{1\leq i\leq m}\sum_{j=1}^{m}b_{ij}^{M}L_{j}\Gamma_{ij}^{*}=2.04>0.$$

In addition, since system (23) has an equilibrium of the type

$$e\left(r,y\right)={\left(0.1545,0.0168\right)}^{T},$$

such that ||e(r,y)||<0.15542, condition 2 of Theorem 1 holds.

Therefore, according to Theorem 1, there exists a unique almost periodic along *r* solution β(r,y) of system (1), uniformly on y∈Θ, such that

(a) $\left||\beta \left(r,y\right)|\right|\leq C_{1},\;C_{1}<0.15542$;
(b) $H\left(\beta ,r_{k}\right)\subset H\left(z,r_{k}\right)$.

In addition, according to Theorem 2, the unique almost periodic solution β(r,y) of system (1) is globally conformably exponentially stable.

## 5. Conclusions

In this paper, the impulsive conformable modeling approach is applied, and an impulsive conformable reaction–diffusion neural network model with distributed delays has been constructed. The almost periodic concept is applied to the proposed model, and the corresponding analysis is performed. The analysis is based on the impulsive conformable Lyapunov technique by means of which efficient sufficient conditions are established for the existence and uniqueness of an almost periodic state of the model. The obtained criteria extend the very few results on the almost periodicity of impulsive conformable neural network systems considering reaction–diffusion terms and distributed delays. The global conformable exponential stability of the almost periodic solution is also studied, and corresponding criteria are proposed. The computational simplicities in the work with conformable derivatives in comparison with the fractional-order derivatives make our results more relevant in applied sciences. The stability results presented can be extended to the integral manifold case considering additional aspects of conformable neural network systems that require further investigation. Some future directions also include developing practical stability or predefined time stability criteria when the Lyapunov-like stability concept cannot be applied. In addition, the effect of uncertain parameters on the qualitative behavior of the proposed model is planned to be studied in future research.

## Data Availability

Data are contained within the article.
